# A Possible Change Process of Inflammatory Cytokines in the Prolonged Chronic Stress and Its Ultimate Implications for Health

**DOI:** 10.1155/2014/780616

**Published:** 2014-06-03

**Authors:** Rui Tian, Gonglin Hou, Dan Li, Ti-Fei Yuan

**Affiliations:** ^1^Department of Psychology, Zhejiang Sci-Tech University, 579 Mailbox, Hangzhou 310018, China; ^2^Department of Psychology, Nanjing Normal University, Nanjing 210097, China

## Abstract

Sustained stress triggers series of changes in the brain and the body. At the early stage of stress, the activated hypothalamus-pituitary-adrenal (HPA) axis and sympathetic nervous system (SNS) axis can upregulate the levels of glucocorticoid (GCs) and catecholamines (CAs), respectively, and then they in turn inhibit the secretion of proinflammatory cytokines directly or indirectly while promoting the secretion of anti-inflammatory cytokines. At the prolonged stage, the sustained activated HPA demonstrates cortisol-resistance. At the same time, the inflammation related transcription pathway, such as nuclear-factor kappa-B (NF-**κ**B) signaling, may be inhibited. Additionally, the inflammatory cytokines mediate a negative feedback regulation on themselves. Collectively, these regulations may increase the proinflammatory cytokines while decreasing the anti-inflammatory cytokines. This may further activate NF-**κ**B and increase the proinflammation cytokines, which in turn reduce the inflammatory responses, contributing to various diseases.

## 1. Introduction

Chronic stress leads to various diseases, mainly through the activation of hypothalamus-pituitary-adrenal (HPA) axis and sympathetic nervous system (SNS) axis, with upregulation of glucocorticoid (GCs) (cortisol in human) and catecholamines (CAs) (noradrenaline and adrenaline), respectively. These secreted molecules then function through separate receptors on different types of cells, including both the nerve cells and immune cells [1–9]. In addition, it is suggested that the chronic stress inhibits proinflammatory cytokines secretion which mediate the cellular immunity, while the stress can also activate anti-inflammatory cytokines secretion that activate the humoral immunity [3–6, 8, 10, 11]. This led to the general hypothesis that chronic stress leads to disease status through immunosuppression [6, 12].

Interestingly, inflammation is a key pathogenic mechanism in many diseases [13–16]. Indeed, neuroinflammation has been recognized as one important aspect in chronic stress-induced depression in both animal models and clinical human patients. Therefore, the effects of chronic stress on inflammation signaling, especially the cytokines, are complicated at different stages of disease in different organs. The present review summarized the roles of cytokines in linking chronic stress to disease.

## 2. Chronic Stress Leads to Downregulation of Proinflammatory Cytokines

Chronic stress exerts the effects on paraventricular nucleus (PVN) and locus coeruleus-noradrenaline center (LC-NA) of hypothalamus as well as brain stem. In response, the hypothalamus secretes corticotropin-releasing factor (CRF) and arginine vasopressin (AVP), which activate the HPA axis and ultimately upregulate GCs from the adrenal cortex. At the same time, the activated locus coeruleus-secreted-noradrenaline can further promote sympathetic-adrenal-medullary (SAM) axis and thereby provoke the release of catecholamines (mainly noradrenaline and adrenaline) from the adrenal medulla to the brain and peripheral blood.

GCs and CAs, respectively, act on glucocorticoid receptors (GR) and adrenergic receptors (*α*-AR and *β*-AR) on the surfaces or in the cytoplasm of immune cells; monocytes and neutrophils are mainly included, which in turn inhibit the secretion of the proinflammatory cytokines, such as IL-1*β*, IL-6, TNF-*α*, and INF-*γ*, while promoting the secretion of the anti-inflammatory cytokines, such as IL-4, IL-10, and IL-13 [7, 17]. The complexes, such as cortisol and GR, enter the nucleus and inhibit the transcription control pathways, including nuclear-factor kappa-B (NF-*κ*B), activator-protein 1 (AP-1), JAK-STAT factors, mitogen-activated protein kinases (MAPKs), signal transducer and activator of transcription 3 (STAT3), and other pathways [12, 18–20], which in turn decrease the proinflammatory cytokines. Besides, motor vagus nerves secrete acetylcholine (ACh), which may also inhibit IL-1*β*, IL-6, and TNF-*α* [7, 21].

**Figure 1:**
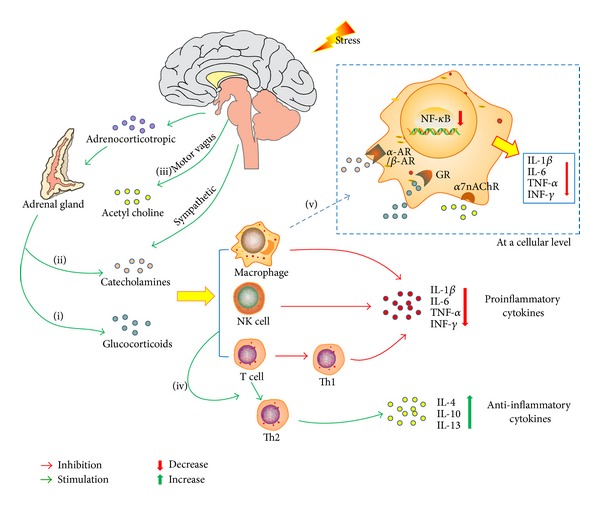
The mechanism of chronic stress acting on inflammatory cytokines. The chronic stress activates the (i) HPA and (ii) SAM axis and, respectively, secretes glucocorticoids and catecholamines hormones, which in turn act on the receptors on the surface or in the cytoplasm of the immune cells, (iii) and, meanwhile, motor vagus fiber also secretes catecholamines and ultimately inhibits the proinflammatory cytokines while promoting the anti-inflammatory cytokines. (iv) Both the glucocorticoids and catecholamines promote the Th1 to Th2 shift, including inhibiting the IL-12 secretion and further boosting the shift. (v) At the cellular level, the signal transduction level, stress hormones inhibit the inflammation related pathways, including NF-*κ*B, and further inhibit the proinflammatory cytokines secretion.

Th1 to Th2 shift is also included in regulating the inflammatory cytokines. Specifically, Th1 cells primarily secrete IL-2, IL-6, TNF-*α*, and INF-*γ*, which activate cytotoxic T cells, natural killer cells, and macrophage and further promote the cellular immunity, whereas Th2 cells secrete a different set of cytokines, primarily IL-4, IL-10, and IL-13, which promote humoral immunity. All of the above pathways can enhance Th1 to Th2 shift [3, 6, 10, 17, 18, 22]. Other studies about the Th1 to Th2 switch suggest that GCs and CAs also act on their classic cytoplasmic/nuclear receptors on APCs to suppress the production of the main inducer of Th1 responses, IL-12, and pathogens invasion also suppresses IL-12 secretion of APCs; so, these all promote the Th1 to Th2 shift; that is, they suppress the proinflammatory cytokines secretion, whereas they enhance the anti-inflammatory cytokines [17, 18, 22] (shown in Figure 1).

In summary, GCs and CAs inhibit the proinflammatory cytokines [23, 24], which are served as the main reason that the activated HPA axis and SNS axis inhibit the proinflammatory factors. In vitro, a negative dose-dependent relationship between GCs and proinflammatory cytokines was found, such as IL-6 and IL-8 [25]. Some researchers found that GCs suppressed IL-1*β*, IL-6, and TNF-*α* production in both the parents caring for a cancer child and the parents of healthy children [23]. At the signal transduction level, stress hormones such as GCs and CAs inhibit the inflammation related pathways, including NF-*κ*B, AP-1, JAK-STAT, MAPKs, and so forth (see above). However, several studies recently indicated that glucocorticoid promotes the secretion of IL-1*β*, IL-6, and TNF-*α*, but they did not rule out other potential signaling pathways downstream of GR activation, which are involved in the inflammatory process [26]. In addition, it has been well established that chronic stress upregulates different hormones, such as CRH, ACTH, GCs, and CAs [1–3, 10], supporting that chronic stress downregulates the proinflammatory cytokines.

## 3. Chronic Stress May Also Increase Proinflammatory Cytokines

There are fewer studies about chronic stress impacting on the cytokines compared with the studies about that on chronic hormones, especially that the cytokines involved are relatively limited, including IL-1, IL-2, IL-6, TNF-*α*, INF-*γ*, EGF, VEGF, TGF-*α*, and so forth, while other cytokines are less involved. This might be because there are few cytokines at high circulating levels in vivo, compared to other stress hormones, especially in asymptomatic individuals. For example, IL-6, whose circulating levels are often higher than those of other cytokines in asymptomatic individuals, is only reliably detectable using high-sensitive assay kits [4].

At present, the research about that has included caregiver stress, job stress and burnout, low socioeconomic status (SES), childhood adversity and life event, lack of social support, loneliness, and so forth. These researches have demonstrated that chronic stress increases the proinflammatory cytokine [1–3, 11, 27–32]. For instance, some researchers have performed meta-analysis to more than 300 studies about chronic stress, and they have found an increased production of IL-6 and INF-*γ* during the chronic stress, compared with the control groups. Although a variety of paradigms are adopted, conclusions are quite consistent [10].

Hence, it is obvious that there is a conflict between the mechanism of chronic stress acting on inflammatory cytokines and the outcomes observed in quite a few studies. These inconsistencies are suggested to be results of the type, duration and intensity of stressors, detection methods, and individual differences [3, 33].

Hans Selye described the “general adaptation syndrome,” activated by an organism in order to overcome various challenges [34, 35]. Although he did not exclude some of the responses to threats which are specific, he considered that most of them are nonspecific; that is, the organism responses activated by different stressors are similar. It was supported by the observations that different stressors could provoke an identical biochemical reaction in the organism, such as cortisol changes. His view has caused considerable controversy, but the main argument has been the definition of the stress (Selye believed that stress is the nonspecific response of the body to any demand mad upon it, with the reaction triad) [36]. Meanwhile, some excellent stress measurement scales, such as the Social Readjustment Rating Scale [37], Perceived Stress Scale [38], the Satisfaction with Life Scale [39], and a modified version of the Profile of Mood States [40], et cetera, whose definition of stressors based on need to activate and adjust the mental and physical resources to deal with, are still widely used to scale the positive and negative stress events and mood states [12, 41]. So, the view that “different psychological stress may cause the identical response” to some extent is accepted.

Whether the chronic stress evoke nonspecific responses or not is not further discussed here, but a basic fact, based on the present studies, is that the proinflammatory cytokines have been observed a consistent increase in many chronic stress studies; for this reason, we believe that identical reactions to a certain extent can be activated by different stressors. Accordingly, excluding the individual differences, what mainly cause the inconsistencies in these studies? Does chronic stress upregulate or downregulate the proinflammatory cytokines? We consider that the prolonged stress can be divided into serial stages, and in each stage the inflammatory cytokines are influenced in different ways. We will mainly discuss this problem in the following.

## 4. The Upregulation/Downregulation Is Stage-Dependent during Chronic Stress

**Figure 2:**
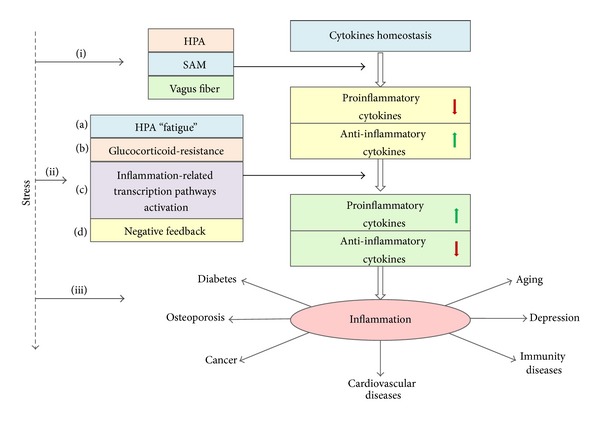
The possible change process of inflammatory cytokines in the chronic stress. (i) The chronic stressor disturbs the cytokines homeostasis through activating HPA axis, SAM axis, and vagus fiber, and they, respectively, promote the secretions of GCs, CAs, and Ach to suppress the proinflammatory cytokines secretion while promoting the anti-inflammatory cytokines secretion. (ii) Sustained stressor exposure changes to upregulate the proinflammatory cytokines and downregulate the anti-inflammatory cytokines through the following four pathways: (a) HPA “fatigue”; (b) glucocorticoid-resistance; (c) the inflammation-related transcription pathways (NF-*κ*B, AP-1, JAK-STAT, MAPKs) activation; and (d) organism negative feedback. (iii) The lasting chronic stress further increases the proinflammatory cytokines, which induces inflammatory response and may ultimately cause various diseases.

We consider that there are three serial stages in the chronic stress, based on all the above (the contradiction between the mechanism of chronic stress impacting on cytokines and the outcomes observed in the current human studies (Figure 2)).

- At the early stage, chronic stress downregulate the proinflammatory cytokines while upregulating the anti-inflammatory cytokines (see above the mechanism in detail).
- Sustained stress may lead to HPA axis “fatigue,” that is, the response of HPA axis to sustained stress can be blunted [18]. Meanwhile, the long-term stress also causes glucocorticoid-resistance; that is to say, with prolonged exposure to stress hormones, glucocorticoid receptors are downregulated and the immune system's sensitivity to the cortisol declines [18, 42, 43]. Evidence of this phenomenon is derived from clinical studies that long-term administration of synthetic glucocorticoid medications to patients with inflammatory disease induces a glucocorticoid-resistance syndrome in which initial therapeutic dosages do not come to offer the effective dose, and a huge number of research support for glucocorticoid-resistance, in which chronic stressors have been shown to diminish the capacity of glucocorticoids to suppress cytokine production, for instance, some researchers have found that the capacity of the synthetic glucocorticoid (dexamethasone) to suppress IL-1*β*, IL-6, TNF-*α*, et cetera, secretion declines [12, 44, 45]. Recent research indicates that multiple mechanisms contribute to this resistance [20], such as repression of GR gene expression by glucocorticoid-induced GR binding to an negative glucocorticoid response element (nGRE) on a GR-NCoR1-histone deacetylase 3-containing repression complex (NR3C1) [46], GR*β* antagonizing the action of GR*α* [47], and phosphorylation of GR by p38 MAPK [48]. Besides, research suggests that epinephrine and norepinephrine can downregulate glucocorticoid receptor expression [7]. So what happened at the cellular levels? The transcription of I kappa B alpha (I*κ*B), induced by glucocorticoid stimulation to block the NF-*κ*B activation, is diminished with the blunted HPA axis and glucocorticoid-resistance. Accordingly, the inflammation related pathways are activated, and they in turn activate the genes responsible for proinflammatory cytokine production [12, 18]. Additionally, the negative feedback of the organism is also accounted for the inflammatory cytokines reregulation [18]. Above all, the proinflammatory cytokines are upregulated, which is consistent with the main results observed in lots of studies, and it implies that the inflammatory cytokines enter the second stage in the change process with chronical stress exposure. Most of the human studies for chronic stress may be at this stage, for the initial of the studies is not from the onset of the stress exposures; on the contrary, they might be constantly or frequently in the stressful conditions before they participated in the studies. Other support for the assumption comes from numerous studies demonstrating that lots of the subjects even show the depressive-like behavior [23, 49–52]. Furthermore, the depressive-like behavior can be induced by the proinflammatory cytokines (see below) [52, 53].
- The second stage is followed by the next stage, unless the sustained stress exposure is removed. The lasting impacts of HPA axis “fatigue,” glucocorticoid-resistance, and activated NF-*κ*B on cytokines cause the proinflammatory cytokines to increase further, which to a certain level induce inflammatory response. In addition, IL-1*β*, IL-6, TNF-*α*, and other cytokines in turn activate NF-*κ*B, in which the mechanism includes the proinflammatory cytokines leading to phosphorylation and loss of I-*κ*B [54], oxidative stress, and proinflammatory cytokines inducing histone acetylation and NF-*κ*B/AP-1 activation [55]. All the steps induce continued increased proinflammatory cytokines, and finally inflammation, which may induce various diseases.

In these current days, the second and the third stage in our presentation, to some extent, equal the “low-grade inflammation” in some research. This “low-grade inflammation” is not the acute inflammation accompanies acute inflammation symptom, such as fever, swelling, and acute infection, but a systematic low-grade inflammation with a longer term phenomenon and an increase in the circulating levels of inflammatory markers, and this state is more sensitive and susceptible to acute stress and other stimuli [56, 57].

## 5. From Inflammation to Diseases

Increasing amounts of data suggest that inflammatory responses have an important role in the pathophysiology of a variety of diseases, including depression, diabetes, cardiovascular disease, and cancer.

Inflammation is characterized by a cellular response whereby the proinflammatory cytokines of the innate immune response promote the expression and release of chemokines and cellular adhesion molecules (CAM) in the local environment. And inflammatory response related immune cells include macrophages, neutrophils, and NK cells, as well as the activated T-helper and T-cytotoxic cells, which are recruited by the proinflammatory mediators to the inflammation region. The proinflammatory mediators also enter the peripheral circulation and promote the liver cells to produce acute-phase reactive proteins (such as C-reactive protein, serum amyloid A, Haptoglobin), and activate the HPA axis, interact with the neural transmitters, and induce behavioral changes: the “sickness behavior” (such as fatigue, depression, and cognitive dysfunction). And all the above reactions subserve the metabolic demand of inflammation [30, 31, 58]. At the cellular levels, NF-*κ*B is activated by kinds of pathways (such as Toll-like receptors, IL-1, el at.) to boost the proinflammatory mediators secretion, including proinflammatory cytokines (IL-1*β*, IL-6, TNF-*α*, IFN-*γ*), chemokines, CAM, and APP [31].

### 5.1. Inflammation and Depression

The proinflammatory cytokines in the peripheral blood go through the weak region of blood-brain barrier or by their specific transport proteins on the brain endothelial cells (the circumventricular organs) or transmit the signals to the specific regions of the brain by the vagus nerve fibers [31]. In the CNS, the proinflammatory cytokines alter the metabolic processes of neurotransmitters, such as serotonin (5-ht) and dopamine (DA) [59], whose secretion suppression and the reuptake block take a role in the pathogenesis of depression and provide advices to the therapy. Then, the proinflammatory cytokines activate the CRH of the PVN and upregulate ACTH and cortisol, and abundant of studies suggest that the overexpression of CRH is the key link between the chronic stress and depression [60]. Besides, the proinflammatory cytokines disrupt synaptic plasticity through altering the relevant growth factors, such as brain-derived neurotrophic factor (BDNF) [61]. And many evidences come from clinical treatment for infectious diseases, in which they present the IFN-*α*-induced depressive symptom, and IFN-*α* is the inducer of IL-6, TNF-*α*, and IL-1*β* [31].

### 5.2. Inflammation and Diabetes

Diabetes is considered as a kind of inflammatory and metabolic diseases, so the overproduction of IL-6, TNF-*α*, and IL-1*β* has been observed in people with diabetes [62, 63]. The mechanism of the inflammatory response leading to diabetes is not fully described, while accumulating data support that the lack of TNF-*α* or inhibiting its receptor induces the increased sensitivity of insulin [62]. Recent studies also suggested that the inflammatory cytokines, including TNF-*α*, could induce insulin-8 resistance [62, 64]. The mechanism of the insulin-resistance involves the activated NF-*κ*B pathway and several relevant serine/threonine kinases, such as c-Jun N-terminal kinase (JNK) and inhibitor of NF-*κ*B (IKK) [65]. In fact, insulin works as proinflammatory factor, and it is involved in the anabolic pathway, whereas the basic inflammatory response favors a catabolic state and suppresses anabolic pathways, such as the insulin signaling pathway [58]. Additionally, the beta cells, which secrete insulin, can be disordered by activated NF-*κ*B, or damaged by inducible nitric oxide synthase (iNOS), which is stimulated by the proinflammatory cytokines [66].

### 5.3. Inflammation and Cardiovascular Disease

Cardiovascular diseases, including atherosclerotic heart disease, coronary heart disease (CHD), congestive heart-failure (CHF), and other cardiovascular diseases, have been associated with elevated levels of IL-1*β*, IL-6, TNF-*α*, and IFN-*γ* [67–69]. These proinflammatory cytokines are believed to contribute to atherosclerotic plaque formation and cardiac irritability. The atheroma is preceded by a fatty streak, an accumulation of lipid-laden cells, macrophages, and some T cells, beneath the endothelium. Then the activated macrophages lead to the release of inflammatory cytokines, chemokines, oxygen and nitrogen radicals, and other inflammatory molecules and, ultimately, to inflammation and tissue damage, which promote the atherosclerotic plaque formation. Besides, the activated NF-*κ*B induces cardiac hypertrophy [67, 69].

Additionally, inflammatory processes are well known to contribute to the onset and progression of immune diseases, including allergic diseases, autoimmune diseases, and infections; there has been increasing data suggesting that inflammatory responses take a role in other diseases. For instance, they may induce osteoporosis. The proinflammatory cytokines can block the vitamin D receptors to disturb the role of vitamin D in the bone formation, and meanwhile, the activated NF-*κ*B promotes osteoclast activity and bone reabsorption [70]. Besides, inflammation signaling pathways, such as NF-*κ*B, have been implicated in the neoplastic process, for they play a role in the tumor cells growth and proliferation as well as in resistance to chemotherapeutic agents [13]. Finally, the concerns about the relationship between inflammation and ageing have been raised in the current years. IL-6, TNF-*α*, and IL-1*β*, and other proinflammatory cytokines are overexpressed in variety of age-related diseases and are positively associated with age. Research suggests that the elevated IL-6 levels lead to high mortality in the elderly [71]. Additionally, IL-6 can inhibit the activity of telomerase and speed up to shorten the length of telomere, which might be associated with premature ageing of cells [1, 72].

## 6. Summary

In conclusion, there is a serial of changes in the proinflammatory cytokines under the sustained stress. At the early stage, chronic stress activates the HPA axis, SAM axis, and the vagus fiber, which in turn secrete GCs, CAs, and Ach to disturb the inflammatory cytokines in their homeostasis and downregulate the proinflammatory cytokines while upregulating the anti-inflammatory cytokines; in the second stage, the lasting stress exposure induce HPA “fatigue,” glucocorticoid-resistance, NF-*κ*B activation, and the negative feedback, which in turn promote the proinflammatory cytokines; in the third stage, the continued stress further increases the proinflammatory cytokines and ultimately cause inflammation, which may induce various diseases.
